# Reliability and Accuracy of MRI in Orthopedics: A Survey of Its Use and Perceived Limitations

**DOI:** 10.1177/1179544119872972

**Published:** 2019-09-05

**Authors:** Andrew Hong, Joseph N Liu, Anirudh K Gowd, Aman Dhawan, Nirav H Amin

**Affiliations:** 1 School of Medicine, Loma Linda University, Loma Linda, CA, USA; 2 Loma Linda University Medical Center, Loma Linda, CA, USA; 3 Wake Forest University Baptist Medical Center, Winston-Salem, NC, USA; 4 Penn State Health Milton S. Hershey Medical Center, Hershey, PA, USA; 5 Veterans Affairs Loma Linda Healthcare System, Loma Linda, CA, USA

**Keywords:** MRI, survey, diagnostic utility, knee, shoulder, reliability, accuracy, orthopedics

## Abstract

Over the past decade, the use of magnetic resonance imaging (MRI) as a diagnostic tool has been increasing significantly in various fields of medicine due to its wide array of applications. As a result, its diagnostic efficacy and reliability come into question. Specifically, in the field of orthopedics, there has been little discussion on the problems many physicians face while using MRIs in practice. To gauge the perceived limitations of MRI, we designed a decision analysis to analyze the utility of MRIs and estimate the number of inconclusive MRIs ordered within an orthopedic practice to explore potential alternative avenues of diagnosis. A survey of 100 board-certified practicing orthopedic surgeons given at 2 national conferences was designed to assess the value, reliability, and diagnostic utility of MRIs in preoperative planning in shoulder and knee surgery. Of those surveyed, 93% reported that there was believed to be a problem with the accuracy of an MRI in the setting of a prior surgery and/or if previous hardware was present specifically pertaining to the knee or shoulder. The most common indications of concern regarding knee or shoulder MRI reliability among this sample group were previous patient hardware (19%), a previous surgery (16%), and a chondral defect (11%). In addition, when asked how many MRIs were believed to be inconclusive based on previous surgery/hardware alone in the last 6 months of practice, an average of 19 inconclusive MRIs was reported. This study summarizes some of the concerns of MRI use in the orthopedic community and attempts to add a unique perspective on the attitudes, decision-making, and apparent economic problems that they face as well as uncover specific instances where MRIs were determined to be unreliable and incomplete in aiding the diagnosis and treatment algorithm.

## Introduction

As medical technology increases in sophistication and ease of use, the usage of these instruments has risen drastically. According to the Organization for Economic Cooperation and Development (OECD),^1^ the United States carried out approximately 89.1 magnetic resonance imaging (MRI) scans per 1000 people in 2006 and 106.8 MRI scans per 1000 people in 2013. The number of MRI scans more than doubled from 2000 to 2013 in the United States. Despite this, there has been some debate regarding the clinical value of MRI within orthopedics. The study possibly highlights the overuse of the MRI for diagnosis and value in treatment. Given the rising popularity of MRI within orthopedic clinical practice, its must be critically assessed to ensure that ordering this additional diagnostic tool is still providing value.

Song et al^2^ evaluated 185 knee MRIs for utility and determined that 43% were arguably useless, 18% were equivocal, and 39% were useful. Another study found an overordering of knee MRI in patients with radiographically obvious end-stage knee osteoarthritis.^3^ This study argues that nonphysicians and nonacademic practices were more likely to order unnecessary MRIs, which may highlight an educational disparity between orthopedic practitioners,^3^ Similarly, within a population of patients with hip arthritis, referral physicians were found to order unnecessary MRIs in 15.4% of patients, which equates to an estimated 330 to 440.5 million dollars.^4^ Alternatively, overuse of MRIs in patients with adhesive capsulitis was found to have a high false-positive interpretation of rotator cuff pathology which was more prevalent in radiologists than surgeons.^5^ Although MRIs haven been proven to be very useful in diagnosing certain pathology, there are many situations where they seem to be more inconclusive or even very misleading and thus raises the question of when MRI should be used. It is apparent that the orthopedic community sees many shortcomings of the MRI.

To gauge the perceived limitations of MRI, we designed a survey to analyze the utility of MRIs and estimate the number of inconclusive MRIs ordered within an orthopedic practice to explore potential alternative avenues of diagnosis. Due to the increasing use of MRIs and the arguable paucity of information that MRIs provide when diagnosing certain conditions, we surveyed 100 orthopedic physicians regarding the challenges and limitations they face while using MRI for preoperative diagnosis and planning for shoulder and knee surgeries. The findings of this study must be balanced with the dynamic growth of MRI technology with respect to 3-dimensional (3D) reconstruction and modeling of anatomy for 3D printing. Patient-matched instrumentation is frequently being used in orthopedics, particularly joint arthroplasty, to facilitate surgery by accounting for patient anatomy.^6-8^ Interestingly, one study suggested that MRI is more accurate than reconstructions performed by computed tomography.^9^ In anticipation of this technology and increasing demand for MRI, the surgeon’s perspective regarding the present state of MRI use in orthopedic practice is even more important. This article will summarize and discuss the findings of what was surveyed regarding MRI use in practice.

This study adds another perspective on the decisions, attitudes, and shortcomings regarding MRI use in an orthopedic setting and uncovers situations in which the use of MRI was incomplete or questionable in aiding in the diagnosis. Also, it adds new information on the challenges in treating patient with unclear MRI findings while shedding light on the certain scenarios in which the MRI is not as helpful in aiding in the diagnosis and treatment of the patient. It is the first study to date that validates the concerns of MRI use from an orthopedic surgeon’s perspective.

## Methods

This study is a cross-sectional survey analysis to gauge the inconsistencies and challenges of using an MRI in preoperative planning and care of shoulder and knee injuries. A survey was constructed by the senior authors of this study to assess the perspective of orthopedic surgeons on use of MRI. General guidelines were used in constructing the survey to minimize bias.^10^ The survey was composed of 2 sections: one quantifying the surgeon’s level of training, and the other focused on assessing the surgeon’s perspective on MRI. In total, 13 questions detailed closed-ended questions with finite or numeric responses, whereas 2 were open-ended and outlined specific scenarios (Table 1).

**Table 1.** Questions asked on survey.

General characteristic survey questions asked	1. What was most recent year medical training was completed? 2. Any fellowship training completed? 3. If applicable, any fellowship training completed? 4. If applicable, any sub-specialty training completed?
MRI-related survey questions asked	1. Roughly how many patients do you see in a month? 2. Is there any perceived problem with the accuracy of an MRI in the settings of: prior surgery and/or if previous hardware was present in the patient? 3. How many MRIs were believed to be inconclusive in last 6 months based on prior surgery or internal hardware? 4. Which indications/situations were most concerning in terms of the reliability of MRI? 5. What is the approximate number of MRIs believed to be inconclusive in last 6 months based on listed indications of concern? 6. What percentage of patients with inconclusive MRIs going to the OR? 7. Is an intra-articular view prior to surgery preferred over an MRI? 8. How much time are you willing to be spent to perform a procedure? 9. How much reimbursement was expected per case? 10. What is the average time spent reviewing MRI results with patient? 11. Is an MRI owned in your practice?

Abbreviations: MRI, magnetic resonance imaging; OR, operating room.

The survey was distributed at 2 national meetings organized by the American Academy of Orthopedic Surgeons (AAOS) on March 1, 2017, and the American Orthopaedic Society for Sports Medicine (AOSSM) on July 7, 2017. Specifically, orthopedic surgeons, regardless of sub-specialty, were the target group of this survey, and it contained questions used to identify problems with MRI in their daily practice. Survey questions were previously printed on paper and were handed out randomly to 190 orthopedic surgeons in total between both conference meetings. In total, 100 orthopedic surgeons’ responses were completed and received. Incomplete surveys were not included in the study.

The participants were asked how much time they were willing to spend in surgery (#8 in Table 1) to gauge their optimal time commitment to perform surgery based on MRI results. The numbers were collected, and the mean, range, and mode were determined where applicable. The results and figures are discussed and summarized below.

**Figure 1.**
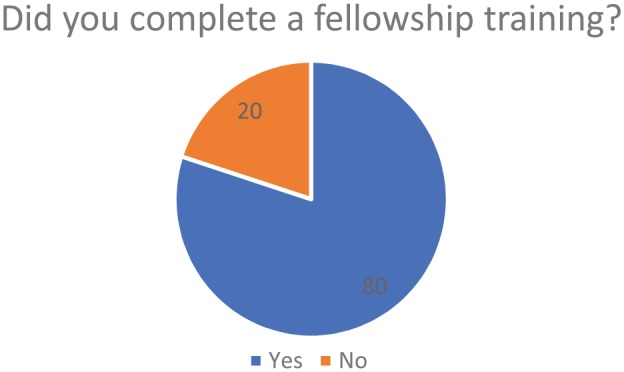
Percentile of study population that completed fellowship training.

**Figure 2.**
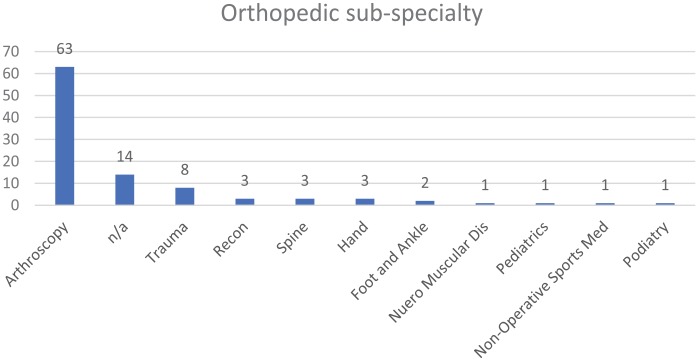
Sub-specialty distribution of the orthopedic surgeons surveyed.

**Figure 3.**
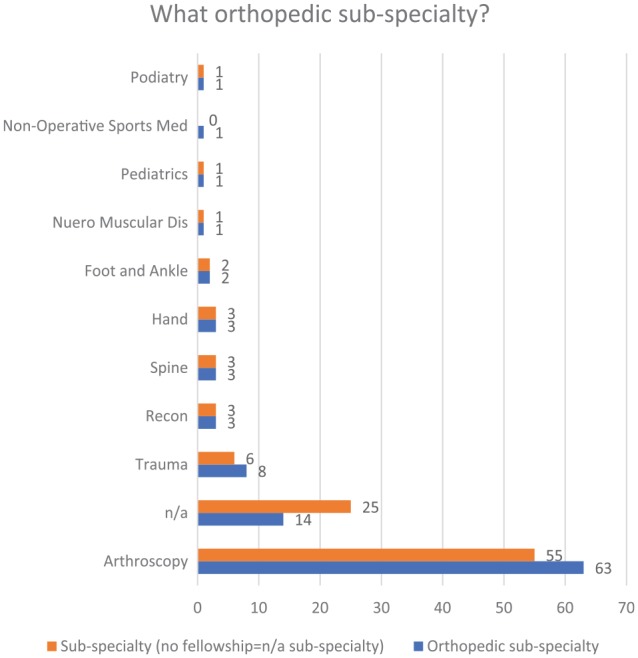
Distribution of orthopedic sub-specialties.

From the 100 orthopedic surgeons’ survey data that was used, the average year in which medical training was last completed was the year 2000, with a range from years 1960 to 2016. Of the 100 participants, 80 participants stated they completed a fellowship (Figure 1). The sub-specialties included arthroscopy (63%), no sub-specialty (14%), trauma (8%), reconstructive (3%), spine (3%), hand (3%), foot and ankle (2%), neuromuscular disease (1%), pediatrics (1%), nonoperative sports medicine (1%), and podiatry (1%) (Figures 2 and 3). On average, 286 patients were seen a month with a range of 25 to 1600 patients.

## Results

**Figure 4.**
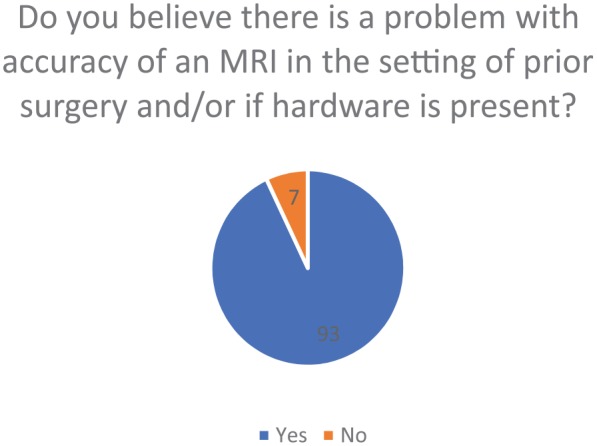
Percentile demonstrating problems with the accuracy of an MRI in the setting of prior surgery or if hardware was present. MRI: magnetic resonance imaging.

When an MRI was obtained in a patient that had a previous surgery or previous hardware (ie, plate, screws, etc), 93 out of 100 participants believed that there was a problem regarding the reliability and diagnostic value that the MRI provided in these specific patients, whereas the remaining 7 did not believe there was a problem (Figure 4). Regarding the number of inconclusive MRIs in the past 6 months based on prior surgery or hardware, an average of 19 inconclusive MRIs was reported with a range of 0 to 300 inconclusive MRIs.

**Figure 5.**
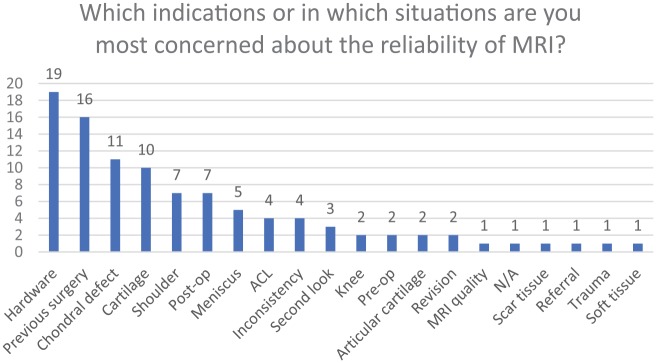
Distribution of the main indications or situations that was of concern about the reliability of an MRI. MRI: magnetic resonance imaging.

**Figure 6.**
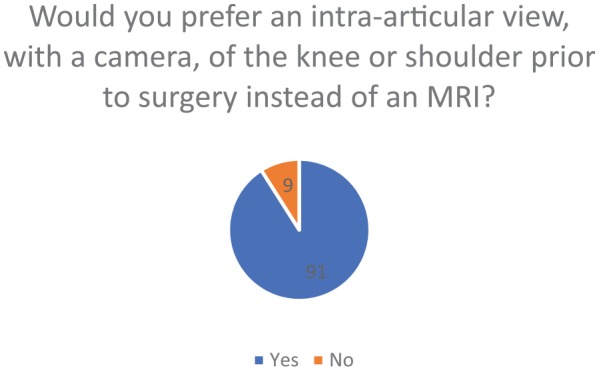
Percentile of surveyed that preferred an intra-articular view as opposed to an MRI prior to surgery. MRI: magnetic resonance imaging.

**Figure 7.**
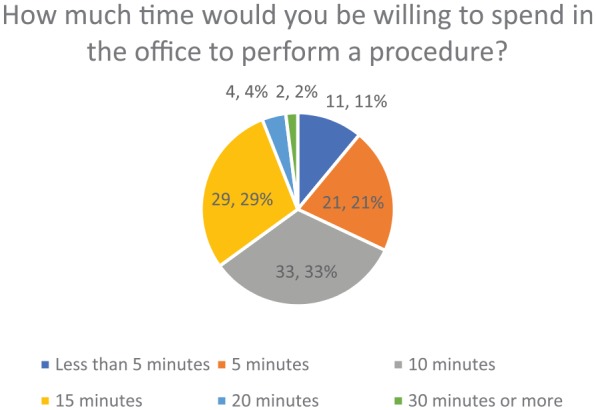
Distribution of how much time was willing to be spent in office for a procedure.

**Figure 8.**
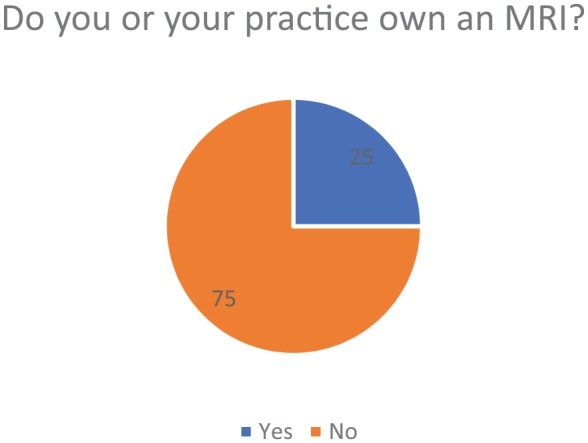
Percentile of practitioners that do not own an MRI in their practice. MRI: magnetic resonance imaging.

The top 4 responses regarding the indications or situations that are most concerning regarding the reliability of an MRI are as follows: previous hardware (19%), previous surgery (16%), chondral defects (11%), and cartilage (10%) (Figure 5). The least concerning indications are as follows: having a second look (3%), and scar tissue, referral, trauma, and/or soft tissue (1%) (Figure 5). To show a general picture of how many MRIs are given and subsequently provided no useful diagnostic value based on the indications above, orthopedic surgeons reported an average of 15 MRIs that were believed to be inconclusive within the last 6 months, with a range of 0 to 150 MRIs deemed inconclusive (mode = 10). We found that an average of 45% of the patients with an inconclusive MRI went to the operating room (OR), with a range of 0% to 100% going to the OR (mode = 50%). Most of the participants (63% being arthroscopy specialists) preferred an intra-articular view of the knee or shoulder with an arthroscope as opposed to an MRI prior to surgery (91%) (Figure 6). Figure 7 shows that 83% of participants were willing to spend only 5 to 15 minutes in the office to perform a procedure. The average reported reimbursement expected per in-office case was US$600, with a range of US$0 to US$2000 per case. Furthermore, the average time in weeks between the first patient visit and reviewing the MRI results with the patient was 2 weeks, ranging from 1 to 20 weeks. In all, 25% of participants reported that their practice owns an MRI, whereas 75% of participants said that their practice did not own one (Figure 8).

## Discussion

In orthopedics, as well as in many of its sub-specialties, the use of an MRI has been a “gold standard” in aiding diagnoses. Although arthroscopy was the most common orthopedic sub-specialty surveyed (63%), there were 9 other sub-specialties surveyed as well as nonspecialized orthopedic surgeons. Thus, in many represented fields of orthopedics, there is a general consensus that MRIs pose problems with accuracy and reliability in patient care and may not be actually helpful in establishing certain diagnoses. Most of the group surveyed believed that there was a problem with the accuracy of an MRI in the setting of a prior surgery and when hardware was present in the patient. The majority (91%) also preferred an intra-articular view of the knee or shoulder prior to surgery as opposed to using an MRI, which indicates that many orthopedic surgeons gain much more diagnostic value with intra-articular views when determining if surgery is necessary. Although the number of MRIs obtained in a month was not surveyed, there was an average of 286 patients seen per month with a resulting average of 19 MRIs in the past 6 months based on prior surgery or hardware alone that were deemed to be inconclusive or of little diagnostic value. The findings of this study suggest an overuse of MRI within orthopedic surgery in that often times this imaging provided little clinical value. This would suggest a need for surgeons, particularly those beginning practice, to increasingly prioritize history-taking, physical examination, and radiographs in management of orthopedic complaints rather than using advanced imaging.

MRI is a powerful diagnostic tool with a multitude of protocols to aid in the detection of soft tissue pathology. Yet, this technology is costly, and indications for its use must be carefully considered. Among the indications and situations that were of most concern regarding the reliability of MRI, hardware was the highest reported reason (19%). The second most reported reliability concern was previous surgeries (16%). Recent advances in MRI technology have made available common protocols for metal artifact reduction sequences.^11^ It is unclear whether surgeons or referring providers use these protocols; however, several studies have demonstrated its capability in assessing intra-articular changes within metal-on-metal or ceramic-on-polyethylene arthroplasties.^12,13^ Additional study may be used to determine the popularity of using these sequences within orthopedic practice as well as the additional cost utility. Diagnoses of chondral defects were the next most reported concern (11%) on MRI. It was found that chondral defects decreased the sensitivity and accuracy of MRIs.^14^ This is because chondral defects are not as easily visible and distinguishable on MRI, so diagnosing these conditions with MRI is challenging and offers little value. Cartilage changes (10%) were also in the top reported areas of concern when using MRI. For similar reasons as in chondral defects, cartilage changes as well as articular cartilage changes (2%) pose significant concern about the reliability of MRI. According to Altinel et al,^15^ degenerative knee pathology was very difficult to determine solely by MRI, and although MRIs provided some degree of information, it was not of much diagnostic value. The shoulder (7%), postop (7%), meniscus (5%), and anterior cruciate ligament (ACL; 4%) all pose a concern for MRI use as well. Inconsistent use of MRI (4%) was another reported concern about MRI reliability. If the physician is unsure in the reasoning for ordering an MRI, it appears the value is more unreliable, similar to published studies.^14^ Referring providers or ancillary staff may order MRI when one is not needed; however, it is important for the specialty surgeons to educate these providers on proper indications as to avoid excess expenses.^3^ These characteristics alone merit further research and study into the protocol and guidelines for using MRI as a tool in diagnosis.

From an economic perspective, it was surveyed that the average in-office reimbursement for our select group of surgeons was on average US$600 per case. This is a relevant point to make when taking into account the increased use of MRI that produces little information about the patients’ condition. The fact that 75% of the physicians surveyed do not own an MRI in their practice may or may not further increase the cost of MRI use if they are sent to other places. Recently, McMillan et al^16^ reported that reimbursement of hospital-based MRI-averaged U$1590 among 5 regions within the United States. Of note, nearly half (45%) of surgeons surveyed would take a patient with an inconclusive MRI to the operating room. Given the significant financial burden of inconclusive MRIs pose on the health care system, it raises the question of alterative options to address pathology prior to the operating room. In addition, MRI may be used as a confirmatory test in cases where history, physical examination, and radiography highly suspect a specific diagnosis. However, it is important to highlight the possibility of false-positive diagnoses with MRI readings, which may further obscure patient management.^5^ Alternatively, many incidences may present itself where MRI may be appropriate, when previously not part of the diagnostic algorithm. Two studies have demonstrated superiority of MRI over radiography in early diagnosis of both HLA-positive and HLA-negative spondyloarthropathies.^17,18^ Orthopedic surgeons must therefore emphasize an up to date understanding of indications to use MRI effectively. Additional value in MRI may be created from the possibility of creating patient anatomical reconstructions for additional modeling.^19^ In cases where MRI would not normally be ordered, doing so will allow for the printing of 3D prostheses.^20-23^ The clinical benefit and utility of these prostheses is yet to be investigated, but they offer a new avenue to provide patient-specific care.

Another aspect to point out is the time spent on reviewing an MRI with a patient that could have otherwise been used in other aspects of their assessment and treatment plan. The average amount of time spent between the patient’s first visit and their review of the MRI results with the physician was 2 weeks with a range of 1 to 20 weeks from our surveyed group. Furthermore, many patients find MRI uncomfortable and time-consuming. In our current health care system, doctors are finding themselves with more documentation requirements and less time with patients. It is possible that the overuse of MRI may add to the problem of time constraint that physicians face. It was also found that most of the participants in our surveyed group (62%) were willing to spend only about 10 to 15 minutes to perform an in-office procedure. If many surgeons prefer not to spend a lot of time in surgery, then the accuracy of preoperative diagnosis using MRI must be more conclusive than not. This survey has brought to light many relevant questions and concerns regarding MRI use and should merit further discussion and research into its actual impact in patient care and physician diagnoses.

Limitations of the study are a small survey of a 100 orthopedic surgeons regarding knee and shoulder surgeries. However, it helps shed some light onto the growing challenges in health care community regarding cost and utility of an MRI. A survey further exploring how frequently MRIs are given would be useful for future understanding. Additional data regarding the protocols typically ordered, accessibility to advanced MRI protocols, and proportion of MRIs ordered by nonphysician providers would be beneficial for study. Further exploration of how many MRIs were ordered over a longer period of time would have added more insight as well. Another significant limitation of this study was relying solely on the recall of the participants to report the numbers of MRIs performed and patients seen in their practice. A more thorough chart review to obtain these numbers could reduce this bias.

## Conclusions

With the advancement of medical technology comes the intricacies of its use and application. Although widely used and accepted technologies such as the MRI have been proven to be very effective in many physicians’ diagnoses, there are also situations where this may not be the case. With the increasing rates of MRI use in medical practice today, questions arise regarding its utility, reliability, and accuracy in specific cases. It is apparent that the orthopedic community sees many shortcomings of the MRI, especially when it involves situations of prior surgery, hardware, or chondral and cartilage defects. Although there is no clear-cut answer as to what the best alternative would be, this survey as well as other studies has shown that an MRI has questionable reliability in a variety of cases. Further study is necessary to determine the accuracy of MRI with different pathologies and to establish specific guidelines on when the use of MRI is best suited for the diagnosis of certain conditions.
